# New and Old Anti-CD20 Monoclonal Antibodies for Nephrotic Syndrome. Where We Are?

**DOI:** 10.3389/fimmu.2022.805697

**Published:** 2022-02-11

**Authors:** Biswanath Basu, Andrea Angeletti, Bilkish Islam, Gian Marco Ghiggeri

**Affiliations:** ^1^ Division of Pediatric Nephrology, Department of Pediatrics, Nilratan Sircar (NRS) Medical College and Hospital, Kolkata, India; ^2^ Division of Nephrology, Dialysis, Transplantation, IstitutoGianninaGaslini Istituto di Ricovero e Cura a Carattere Scientifico (IRCCS), Genoa, Italy; ^3^ Laboratory on Molecular Nephrology, IstitutoGianninaGaslini Istituto di Ricovero e Cura a Carattere Scientifico (IRCCS), Genoa, Italy; ^4^ Department of Pediatrics, Nil Ratan Sircar Medical College and Hospital, Kolkata, India

**Keywords:** nephrotic syndrome, anti-CD20 antibodies, rituximab, ofatumumab, obinutuzumab, daratumumab, glomerulonephritis, immune dysfunction

## Abstract

Nephrotic proteinuria is the hallmark of several glomerulonephritis determined by different pathogenetic mechanisms, including autoimmune, degenerative and inflammatory. Some conditions such as Minimal Change Nephropathy (MCN) and Focal Segmental Glomerulosclerosis (FSGS) are of uncertain pathogenesis. Chimeric anti-CD20 monoclonal antibodies have been used with success in a part of proteinuric conditions while some are resistant. New human and humanized monoclonal anti-CD 20 antibodies offer some advantages based on stronger effects on CD20 cell subtypes and have been already administered in hematology and oncology areas as substitutes of chimeric molecules. Here, we revised the literature on the use of human and humanized anti-CD 20 monoclonal antibodies in different proteinuric conditions, resulting effective in those conditions resistant to rituximab. Literature on the use of human anti-CD 20 monoclonal antibodies in different proteinuric diseases is mainly limited to ofatumumab, with several protocols and doses. Studies already performed with ofatumumab given in standard doses of 1,500 mg 1.73m^2^ suggest no superiority compared to rituximab in children and young adults with steroid dependent nephrotic syndrome. Ofatumumab given in very high doses (300 mg/1.73m^2^ followed by five infusion 2,000 mg/1.73 m^2^) seems more effective in patients who are not responsive to common therapies. The question of dose remains unresolved and the literature is not concordant on positive effects of high dose ofatumumab in patients with FSGS prior and after renal transplantation. Obinutuzumab may offer some advantages. In the unique study performed in patients with multidrug dependent nephrotic syndrome reporting positive effects, obinutuzumab was associated with the anti-CD38 monoclonal antibody daratumumab proposing the unexplored frontier of combined therapies. Obinutuzumab represent an evolution also in the treatment of autoimmune glomerulonephritis, such as membranous nephrotahy and lupus nephritis. Results of randomized trials, now in progress, are awaited to add new possibilities in those cases that are resistant to other drugs. The aim of the present review is to open a discussion among nephrologists, with the hope to achieve shared approaches in terms of type of antibodies and doses in the different proteinuric renal conditions.

## Introduction

Nephrotic Proteinuria is the hallmark of several renal diseases characterized by age dependent peculiarities and different pathogenesis. In adulthood, nephrotic proteinuria is generally due to autoimmune or degenerative diseases, such as Membranous Nephropathy (MGN) and Lupus nephritis (LN) or Myeloma. Pathologies of uncertain origin, such as Minimal Change Nephropathy (MCN) and Focal Segmental Glomerulosclerosis (FSGS), occur most frequently in children and young adults and account for above 90% of cases of nephrotic syndrome under 24 years.

Prednisone is the first line therapy in many cases, however, prevalently patients with MCN may develop steroid dependence (SDNS) requiring steroid sparing-agents to minimize drug-related adverse effects (1–3). Between 10 and 20% of patients have steroid-resistant nephrotic syndrome (SRNS), that requires alternative therapies such as calcineurin inhibitors or mycophenolate and 5% are resistant to all associations (3).

In last decade, clinical trials have shown that rituximab, a chimeric monoclonal antibody targeting the CD20 antigen expressed on B cells (4), may represent an effective treatment in all the spectrum of proteinuric glomerulonephritis in spite of their different origin (5–15). The existence of patients who are resistant to rituximab and others who developed anti-rituximab antibodies following multiple treatments with the drug (16–18) have stimulated the search of novel anti-CD20 molecules (19).

Several monoclonal human or humanized anti-CD20 antibodies have been developed, based on the technology for reshaping therapeutical human antibodies, as described by Riechmann et al. in 1988 (20) and many of them have been already administered in hematology and oncology areas.

In the present review, we will describe the impact and future perspectives of three new anti-CD20 antibodies already approved in different clinical settings (ofatumumab, obinutuzumab, ublituximab) and resulted promising in treatment of proteinuric disease. Depending on the structural aspects and on the number of binding sites, first and second generation anti-CD20 antibodies play different effects on CD20 cell subtypes by direct cytotoxicity, antibody-mediated cytotoxicity (ADCC), phagocytosis (ADCP) or complement-mediated cytotoxicity (CDC) (**Table 1**). Ofatumumab induces a potent stimulus for CDC, whereas obinutuzumab is a powerful activator of ADCC and ADCP and has also a strong direct cytotoxicity, but not a relevant CDC. Overall, fully human and humanized anti-CD20 antibodies demonstrated stronger *in vitro* activities than rituximab. Whether these cellular effects may translate into superior clinical benefits is unknown. The number of studies testing new anti-CD20 antibodies in glomerular diseases has grown in parallel with the expansion of studies in other clinical areas and results from randomized clinical trials are now appearing that may modify therapeutic strategies in a near future.

**Table 1:** Chimeric and humanized anti-CD20 determines different effects on their cell targets depending on their structure, number and extension of the binding sites.

Anti-CD20 Antibodies	Type	ADCC	Direct Cytotoxicity	CDC	ACDP
Rituximab	I	++	+	++	++
Ofatumumab	I	++	+	++++	+++
Obinutuzumab	II	++++	++++	+	++++
Ubituximab	I	++++	++	++	++++

ADCC, antibody-dependent cell-mediated cytotoxicity; CDC, complement-dependent cytotoxicity; ACDP, antibody-dependent cellular phagocitotsis.

+, really low; ++, low; +++, moderate; ++++, high.

Whether human anti-CD20 should be preferred to humanized antibodies and the key question of dosages are main items to be discussed and shared.

## Rituximab: Cell Target and Anti-Proteinuric Mechanism

Rituximab, the first class of anti-CD20 antibody used in renal diseases (21) is a chimeric monoclonal antibody composed of a murine immunoglobulin variable region mounted on a human immunoglobulin G1 heavy chain. It targets the CD20 antigen of B cells that appears early during maturation but not expressed by B cell precursors. Upon CD20 binding, B cells are killed trough different mechanisms, as previously reported. The lowering effects of rituximab on B cells is delayed over time with a median of 6 months, whereas the effect on memory B cells perdures for more than one year.

Reduction of antibodies production by B cells seems the obvious mechanism for the protective effect of rituximab in antibody-mediated renal diseases, such as MGN and LN. It is so far unknown how the drug works in SDNS, not an antibody mediated disease. Lack of clear evidence on mechanisms responsible of SDNS complicates any further evolution on the drug activity (7). Early studies suggested a cross interaction of rituximab with podocyte spingomyelinase-like phosphodiesterase 3b precursor (SMPDL3B) which regulates acid sphingomyelinase (ASMse) in the raft of podocytes and partially co-localizes with synaptopodin, a regulator of thecytoskeleton. In many circumstances the effect of rituximab was associated with depletion of B cells (22), but there are cases of treatment failure despite B cells depletion (23). On the opposite, persistent remission induced by rituximab can be maintained in some patients also after CD19+ recovery (24). Memory B cells have been associated with SDNS disease activity, which may explain the effects of B cell depletion (25). Other immune cells may be involved in rituximab activity, including regulatory T cells (26).

## New Anti-CD20 Antibodies

### Structure and Binding Affinity

**Figure 1:**
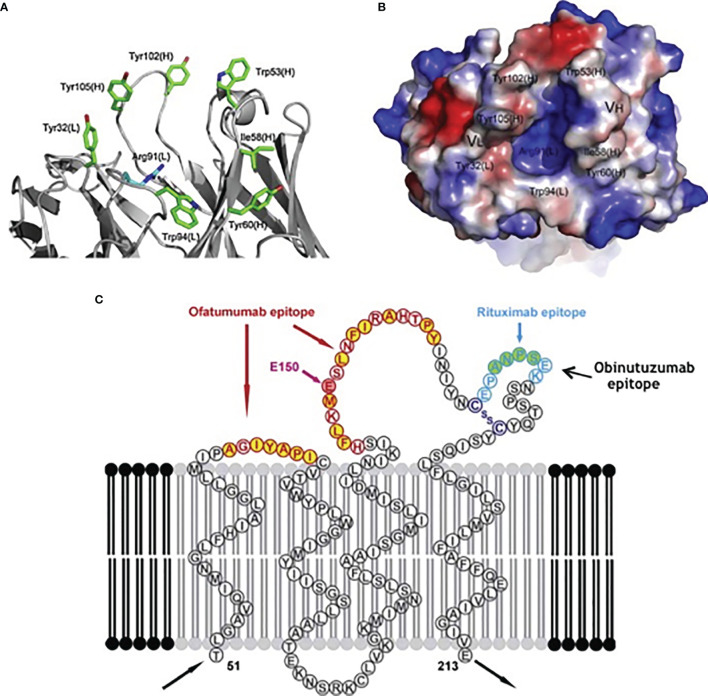
Binding sites for specific targets present in different anti-CD20 monoclonal antibodies: **(A)** details, **(B)** crystalized structure, **(C)** overview.

*Ofatumumab* is a type I humanized anti-CD20 monoclonal antibody that binds the CD20 target through the Fab domain at a distinct epitope respect to rituximab (**Figure 1**) and determines its immune effect through the Fc domain (27). The epitope is closer to the cell surface and the binding site is more extended if compared respect of other anti-CD20 antibodies. Ofatumumab possess a binding site for C1q that mediates an enhanced CDC activity (28).

*Obinutuzumab* is a type II humanized anti-CD20 monoclonal antibody that induces a direct cell death and antibody-dependent cell-mediated cytotoxicity. It has a glyco-engineered Fc region which can enhance binding affinity to the Fc receptor (FcR) on immune effector cells (28, 29). Since the characteristic of type II antibodies is that they do not localize into the lipid rafts, CDC is reduced compared to rituximab and Ofatumumab.

*Ublituximabs* is a chimeric IgG1 monoclonal antibody that recognizes a unique epitope in CD20 and has enhanced affinity for the FcγRIIIa of effector cells and macrophages that mediates increased ADCC and ADCP (30, 31).

### Extra-Renal Fields of Application

Hematolgy is the main area of application of the anti-CD20 antibodies. Ofatumumab has been approved by the FDA for the treatment of multidrug-resistant chronic lymphocytic leukemia (CLL) and has been shown to have efficacy against rituximab-resistant B-cell cancers (32) in combination with other chemotherapeutics (33). Ofatumumab has also been approved as maintenance therapy for the same condition (34). In 2013, Obinutuzumab was approved by FDA for the treatment of CLL after the open-label, three arms trial CLL11 comparing obinutuzumab with rituximab and chlorambucil (35). Similar results were reported in the phase 2 and 3 GALTON trials (36). Efficacy and safety of ofatumumab and obinutuzumab compared with rituximab in different clinical conditions are still controversial. The HOMER randomized study compared the effects of rituximab *vs*. ofatumumab in patients with non-Hodgkin lymphoma (NHL) (37): the trial was stopped early because of futility at the planned interim analysis, indicating non-superiority of ofatumumab. Analysis of results deriving from 11 RCTs comprising 5261 patients with CD20+ NHL showed that ofatumumab, compared to rituximab, had no significant differences in terms of progression-free survival, overall survival and complete response rate, but was inferior in consideration of the overall response rate. Compared with rituximab, obinutuzumab significantly prolonged the progression free survival but it had no improvement on overall response rate, and on complete response rate. Obinutuzumab also increased the incidence of serious adverse effects (OR 1.29, 95% CI 1.13–1.48, *P* < 0.001) (38). Studies *in vitro* strengthened the effectiveness of obinutuzumab in combination with other agents against rituximab resistant cells (39).

## Ofatumumab for Proteinuric Renal Diseases

### Immunoglobulin A Vasculitis With Nephritis

IgAVN is a systemic leukocytoclastic vasculitis characterized by purpuric skin manifestation, usually enteritis and arthritis and frequently glomerulonephritis with IgA deposition in the glomerular mesangium (40). Evidence-based optimal therapy recommendations in cases of IgAVN are not available due to the variability of clinical presentation (41). A recent report described 3 cases treated with rituximab and one with the association of rituximab plus 4 doses ofatumumab (8). Massive B-cell depletion was in this case associated with decrease of proteinuria and stabilization of renal function (**Table 2**).

**Table 2:** Main studies reporting administration of humanized anti-CD20 in nephrotic disease.

Reference	Drug Type	N	F/U (mo)*	Studydesign	Dose	CR (%)
**IgAVN**						
Lundberg S, *Clin Kidney J*, 2017 10 (1):20-26 (8)	Rituximab + Ofatumumab	1	12	Case Report	RTX 375mg/m^2^ + OFA 175mg/m^2^	100
**MGN**						
Sethi S, *K Intern Rep*, 2020 5: 1515-1518 (42)	Obinutuzumab	10	18 (9–24)	Caseseries	N/A	40
**MDNS**						
Ravani P, *J Am Soc Nephrol*, 2021 32 (10):2652-2663 (14)	Ofatumumab	70	12	RCT	1.500 mg/m^2^ (1 dose)	47
Vivarelli M, *Pediatr Nephron* 2017 32 (1):181-184 (43)	Ofatumumab	2	17 (15-19)	Case series	750 mg/m^2^ (1 dose)	50
Fujinaga S, *Pediatr Nephrol*, 2018 33(3):527-528	Ofatumumab	1	5	Case Report	300mg/m^2^	100
**MRNS**						
Basu B, *N Engl J Med* 2014, **370**:1268-1270 (19).	Ofatumumab	5	12	Case series	300mg/m^2^ followed by 5 weekly infusions (2g/m^2^)	80
Wang CS, *Pediatr Nephrol. 2017 32(5):835-841* (44)	Ofatumumab	4	9 (6-13)	Case series	300mg/m^2^ followed by 5 weekly infusions (2g/m^2^)	50
Vivarelli M, *Pediatr Nephrol*, 2017 Jan;32(1):181-184 (43)	Ofatumumab	2	17 (15-19)	Case series	750 mg/m^2^ (1 dose)	50
Bonanni A, *BMJ Case Rep* 2015, 10.1136/bcr-2015-210208 (45)	Ofatumumab	6	12	Case series	375-700 mg/m^2^ (1 dose)	33
Ravani P, *Pediatr Nephrol*, 2020 35(6):997-1003 (46)	Ofatumumab	7	12	RCT	1.500 mg/m^2^ (1 dose)	0
**Post Tx recurrence FSGS**						
Wang CS, *Pediatr Nephrol.* 2017 *32(5):835-841* (44)	Ofatumumab	1	4	Case Report	300mg/m^2^ followed by 5 weekly infusions (2g/m^2^)	100
Colucci M, *Pediatr Nephrol*, 2020 35(2):341-345 (47)	Ofatumumab	2	12	Case series	750 mg/m^2^ (1 dose)	50
Solomon S, *Pediatr Transplant, 2019 Jun;23(4):e13413* (48)	Rituximab + Ofatumumab	1	12	Case Report	RTX 375mg/m^2^ (2 doses) + OFA 2g/m^2^ (2 doses)	100
Reynolds BC, *Pediatr Nephrol*. 2021 Aug 12 (49)	Ofatumumab	7	24	Case series	300mg/m^2^ followed by 5 weekly infusions (2g/m^2^)	44
Kienzl-Wagner K, *Am J Transplant*, 2018 18(11):2818-2822 (50)	Ofatumumab	1	8	Case Report	1150 mg/m^2^ + 1150 mg/m^2^ at 6mo	100
Bernard J, *Pediatr Nephrol*, 2020 35(8):1499-1506 (51).	Ofatumumab	6	10 (8-12)	Case series	300mg/m^2^ followed by 5 weekly infusions (2g/m^2^)	0 (50 PR)

*Data are presented as common follow-up period for all the patients or as median (range).

CR, complete remission; MDNS, multidrug dependent nephrotic syndrome; MGN, membranous glomerulonephropathy; N/A, not available; OFA, ofatumumab; PR, partial remission; RTX, rituximab; RCT, randomized controlled trial; MDNS, multidrug dependent nephrotic syndrome; SRNS, steroid resistant nephrotic syndrome; Tx, kidney transplantation.

### Membranous Nephropathy

In the literature, only one patient affected by MGN was treated with ofatumumab. Podestà et al. (52) described the case of a young male with podocyte phospholipase A2 receptor positive MGN resistant to 4 cycles of rituximab and then treated with the fully human anti-CD20 monoclonal antibody ofatumumab, achieving remission of the NS, without significant side effects. Four doses of ofatumumab were administered over 4 years allowed long lasting maintenance of normal urinary parameters (**Table 2**).

### Childhood Steroid Dependent Nephrotic Syndrome

According to recent case reports and small case series, ofatumumab may induce disease remission in children with SDNS (43, 44) and it was administered in place of rituximab in patients with circulating anti-rituximab antibodies (53).

Positive results stimulated a monocentric randomized clinical trial comparing rituximab and ofatumumab in children with calcineurin dependent SDNS: as main result, we reported that a single dose of ofatumumab was not superior to a single dose of rituximab in maintaining remission in children with steroid- and calcineurin inhibitor-dependent NS (54). After 12 months, the same percent of patients in the ofatumumab and rituximab groups were in remission (i.e. 46 and 47% respectively) then the curve diverged and after 24 months emerged a higher percent of patients in remission in the rituximab *vs*. the ofatumumab group (i.e. 34% and 24% respectively). An ancillary finding was that ofatumumab produced better results in children >16 years than below this age (**Table 2**).

### Childhood Multidrug Resistant Nephrotic Syndrome

Treatment of children with multidrug resistant nephrotic syndrome (MRNS) is a major clinical concern due to a very high probability of evolution to end stage renal failure. The optimal dosing of ofatumumab for renal conditions has not been established yet, especially in children, and studies so far published are mainly limited to case reports and case series. Three papers reporting small case series suggested that the fully humanized anti-CD 20 antibody ofatumumab could be more effective than the chimeric compound in MRNS and encouraged clinical testing. Basu et al. (19) treated 5 children with nephrotic syndrome with well-defined resistance to rituximab, tacrolimus/ciclosporin and cyclophosphamide with high dose ofatumumab (300 mg/1.73m^2^ followed by five infusion 2,000 mg/1.73 m^2^) and observed normalization of proteinuria within 6 weeks. Similarly Wang et al. (44) showed promising results with high dose. Bonanni et al. treated 6 children with the same clinical characteristics with a ‘low dose’ approach (300 mg followed by 700 mg/1.73 m^2^ in two weeks) and observed remission of proteinuria in 2 cases (45). Safety of ofatumumab given in high doses may represent a main problem that requires much attention.

Ravani et al. (46) designed a randomized placebo-controlled trial in children with MRNS comparing ofatumumab administered at hematologic doses (1,500 mg/1.73 m^2^) *vs.* placebo and did not support potential benefits of ofatumumab (**Table 2**).

Based on results of studies using high dose ofatumumab and the negative results with low cumulative dose, there is a need of more safety data. Confirmatory studies on the effect of high doses ofatumumab in patients with MRNS are also needed.

### Post-Transplant Recurrence of Nephrotic Syndrome

The treatment of post-transplant recurrence of nephrotic syndrome is often challenging. Disease recurrence after renal transplantation occurs in around half of cases. Early recurrence is more common in pediatric patients who may present massive proteinuria within hours or days after transplantation; efficacy of therapeutic strategies is often limited.

Ofatumumab has been proposed in treating post-transplant FSGS relapse based on single case reports and small series (44, 47–49). Kienzl-Wagner et al. (50) demonstrated decrease of proteinuria in a patient with second transplant after treating with daily plasma exchange and ofatumumab. At 8 months after kidney re-transplantation graft function was in normal range.

In the largest series involving six children with recurrence of nephrotic syndrome after renal transplantation with failure of previous treatments, ofatumumab demonstrated poor efficacy (51). Four children were treated with the high dose described by Basu (19) and were followed for 10.5 months. No patient achieved a complete remission, half of them had a partial remission and half had no response at all (**Table 2**).

### Cell Monitoring

Human and chimeric anti-CD20 antibodies target the same polymorphonuclear sub-populations. A detailed comparison between ofatumumab and rituximab has been done in the recent randomized study comparing the two drugs in SDNS children (54): the whole B cells compartment was reduced to zero, with minimal differences in the re-population kinetics between ofatumumab and rituximab. Overall, B-cells started to recover after 3-4 months after infusion while Memory B cell, in particular IgM Memory B cells, remained very low during the first 12 months after infusion and then started to regenerate.

T cells were only minimally modified and remained stable during the follow up. A modest increment in the percent concentration of CD3Tcells, CD53 NK and Treg cells in the 6 months following infusion was observed.

## Obinutuzumab for Resistant Glomerulonephritis

### Childhood Multidrug Dependent Nephrotic Syndrome

Only one study used obinutuzumab (single dose 1,000 mg 1.73m^2^) in nephrotic syndrome in combination with sequential administration of the anti-CD38 (plasma cell) monoclonal antibody daratumumab (1,000 mg 1.73m^2^) after 14 days (55). Fourteen patients who relapsed after conventional treatments with prednisone, rituximab and CNI and developed dependency with combination of more drugs (MDNS) were treated with the above association of obinutuzumab and daratumumab and were included in a retrospective analysis: 5 presented recurrence of proteinuria after about 10 months, 9 were in stable remission after 20 months of follow up. The use of 2 monoclonal antibodies at low doses is of interest in consideration of safety. On the other hand, to discern the separate effects of obinutuzumab and daratumumab when given together is not possible; however, the positive results of combining therapy opens new ways in the treatment of nephrotic syndrome and supports the necessity of new studies in the future in those patients who require more than one drug to maintain remission.

### Membranous Nephropathy

In a recent case series, Sethi et al. (42) treated with obinutuzumab 10 adults with MGN, with well-defined resistance to rituximab, tacrolimus and cyclophosphamide. Authors reported that 60% of patients achieved complete or partial remission at 6 months and almost 90% after 12 months of follow up.

### Lupus Nephritis

In MRL/lpr mice, a murine model of Lupus, obinutuzumab resulted more effective in depleting B cells than rituximab (56). A Randomized Controlled Study comparing obinutuzumab and rituximab in subjects with Proliferative Lupus Nephritis (class III and IV) is now in progress: preliminary results are showing that obinutuzumab provides sustained clinical benefit through 2 years compared to rituximab. Results of the phase 2 NOBILITY trial (NCT02550652), comparing the efficacy and safety of obinutuzumab plus MMF with placebo plus MMF in participants with proliferative Lupus Nephritis, also showed that obinutuzumab was well-tolerated with no unexpected safety findings at two years of follow up.

## Safety

### Ofatumumab

Safety of new anti-CD20 monoclonal antibodies is an important issue considering that in many cases with resistance to other drugs, very high doses, particularly of ofatumumab, are required.

Previous observations in patients treated with standard dose of ofatumumab (1,500 mg 1.73 m^2^) indicated an increased respiratory susceptibility when compared with rituximab. Several patients presented bronchospasm and required infusion of the drug in a protected condition (57). The association of nebulized salbutamol in the pretreatment drug schedule resolved almost completely this problem.

Bonanni et al. (57) compared safety of ofatumumab and rituximab in large cohorts of patients (268 *vs* 68 rispectively) showing higher incidence of respiratory symptoms with infusion of ofatumumab. Of note, the retrospective observatory study included infusions administered in the pre-salbutamol era. The results on safety of humanized and chimeric anti-CD20 presented in the randomized trial comparing their effects in SDNS confirmed low negative impact for both compounds (54).

Four of the 10 patients treated with ofatumumab due to recurrence of nephrotic syndrome after kidney transplantation (55) exhibited minor allergic reactions; one patient died of infection as a consequence of multiple factors.

### Obinutuzumab

Three patients treated with obinituzumab presented mild infusion reactions, i.e. vomiting and urticaria and transient neutropenia was observed in other limited subjects (55).

One patient of the membranous series had respiratory symptoms during obinutuzamab infusion and 4 had leukopenia that lasted for more than 3 months in one of them (42).

## Conclusion and Perspectives

The introductions in clinical practice of human and humanized anti-CD20 monoclonal antibodies has represented a break-through for the treatment of proteinuric renal diseases and offer the opportunity to reconsider those clinical conditions resistant or partially responsive to rituximab. A main issue is multidrug resistant FSGS in the pre and post-transplant phases. Looking to a possible future clinical trial, the key questions remain which anti-CD20 antibody and at which dose should be administered. The extremely positive results obtained with the combination of obinutuzumab and daratumumab in patients with severe MDNS, offer the possibility to consider this association also for these patients. The opportunity to use both drugs in medium-low doses probably minimizes side effects and strengthens the necessity of formulation of new therapeutic approaches. Post-transplant recurrence of FSGS is another condition that should be considered for the association of obinutuzumab and daratumumab. Other combinations of daratumumab, e.g. with rituximab, could also be considered.

Obinutuzumab represent an evolution also in the treatment of autoimmune glomerulonephritis, such as MGN and lupus nephritis. Results of randomized trials now in progress are awaited to add new possibilities in those cases resistant to other drugs.

## Author Contributions

BB, AA, BI, and GG contributed to conception and writing of the work. BB and GG revising it critically. All the authors provide approval for publication of the content.

## Funding

The study was supported with public funds granted by the Italian Ministry of Health “*Ricerca Corrente 2021*”.

## Conflict of Interest

The authors declare that the research was conducted in the absence of any commercial or financial relationships that could be construed as a potential conflict of interest.

## Publisher’s Note

All claims expressed in this article are solely those of the authors and do not necessarily represent those of their affiliated organizations, or those of the publisher, the editors and the reviewers. Any product that may be evaluated in this article, or claim that may be made by its manufacturer, is not guaranteed or endorsed by the publisher.
